# The subcortical belly of sleep: New possibilities in neuromodulation of basal ganglia?

**DOI:** 10.1016/j.smrv.2020.101317

**Published:** 2020-08

**Authors:** Harutomo Hasegawa, Richard Selway, Valentina Gnoni, Sandor Beniczky, Steve C.R. Williams, Meir Kryger, Luigi Ferini-Strambi, Peter Goadsby, Guy D. Leschziner, Keyoumars Ashkan, Ivana Rosenzweig

**Affiliations:** a Sleep and Brain Plasticity Centre, Department of Neuroimaging, Institute of Psychiatry, Psychology and Neuroscience (IoPPN), King's College London (KCL), UK; b Department of Neurosurgery, King's College Hospital, London, UK; c Sleep Disorders Centre, Guy's and St Thomas' Hospital, London, UK; d Danish Epilepsy Centre, Dianalund, Denmark; e Aarhus University Hospital, Aarhus, Denmark; f Department of Neuroimaging, IoPPN, KCL, UK; g Pulmonary, Critical Care and Sleep Medicine, Yale School of Medicine, Connecticut, USA; h Università Vita-Salute San Raffaele, Milan, Italy; i NIHR-Wellcome Trust Clinical Research Facility, SLaM Biomedical Research Centre, King's College London, London, UK; j Department of Neurology, Guy's and St Thomas' Hospital (GSTT) & Clinical Neurosciences, KCL, UK

**Keywords:** Basal ganglia, Sleep, Neuromodulation, Memory

## Abstract

Early studies posited a relationship between sleep and the basal ganglia, but this relationship has received little attention recently. It is timely to revisit this relationship, given new insights into the functional anatomy of the basal ganglia and the physiology of sleep, which has been made possible by modern techniques such as chemogenetic and optogenetic mapping of neural circuits in rodents and intracranial recording, functional imaging, and a better understanding of human sleep disorders. We discuss the functional anatomy of the basal ganglia, and review evidence implicating their role in sleep. Whilst these studies are in their infancy, we suggest that the basal ganglia may play an integral role in the sleep-wake cycle, specifically by contributing to a thalamo-cortical-basal ganglia oscillatory network in slow-wave sleep which facilitates neural plasticity, and an active state during REM sleep which enables the enactment of cognitive and emotional networks. A better understanding of sleep mechanisms may pave the way for more effective neuromodulation strategies for sleep and basal ganglia disorders.

## Abbreviations

5-HT: 5-hydroxytryptamine
CM: centromedian nucleus
CNS: central nervous system
ESS: Epworth Sleepiness Scale
GABA: Gamma amino butyric acid
GPi: globus pallidus internus
LC: locus coeruleus
LDT: laterodorsal tegmental nucleus
MCH: melanin concentrating hormone
NE: norepinephrine
NREM: non rapid eye movement
OX-SAP: orexin-2-saporin conjugate
PB: parabrachial nucleus
PD: Parkinson's disease
PLM: periodic limb movements
PPN: pedunculopontine nucleus
PPT: pedunculopontine-tegmental nucleus
PSG: polysomnography
PSQI: Pittsburgh Sleep Quality Index
RBD: REM behaviour disorder
REM: rapid eye movement
RLS: restless legs syndrome
RWA: REM without atonia
SLD: sublaterodorsal nucleus
SNc: substantia nigra compacta
SNr: substantia nigra reticulata
STN: subthalamic nucleus
TMN: tuberomamillary nucleus
VLPO: ventrolateral preoptic nucleus
VTA: ventral tegmental area

## Introduction

Since the first description of the corpus striatum by Thomas Willis in 1664, our conception of the basal ganglia has changed from a collection of static structures to parts of a dynamic network, supporting a diverse range of functions within the central nervous system [1]. The structures comprising the basal ganglia are the corpus striatum, globus pallidus (GP), subthalamic nucleus (STN) and substantia nigra (SN) [2]. The functional organisation of the basal ganglia is highly conserved from the lamprey, suggesting that it serves a fundamental role in vertebrate phylogeny [3]. The basal ganglia have classically been considered in relation to movement disorders [4,5], but their wider role in the integration of sensorimotor, cognitive and affective functions has increasingly been recognised [6,7]. For example, the involvement of the STN in cognitive processing and emotional valence has recently been demonstrated in humans using intracranial recordings [8,9], and the effects of basal ganglia disorders and their effects on non-motor symptoms is well established [10].

Early studies posited a relationship between the basal ganglia and sleep [11,12] but this relationship has subsequently received relatively little attention such that they are almost ignored in recent discussions on the functional neuroanatomy of sleep but see [[13], [14], [15]] for important discussions. It is timely to reconsider the role of the basal ganglia in sleep given emerging insights into the functional anatomy of the basal ganglia [1,16,17] and significant progress in number of clinical interventions that allow intracranial recordings from the human brain during sleep [18]. Does activity in the basal ganglia follow changes instigated by sleep mechanisms in the reticular activating system, or do the basal ganglia play an intrinsic role in regulating the sleep/waking cycle? We review the functional neuroanatomy of the basal ganglia and discuss the implications for the functions and neuromodulation of sleep.

## Functional anatomy of the basal ganglia

In the 1990s, the model of basal ganglia as a series of segregated loops consisting of the ‘direct’ and ‘indirect’ pathways gained popularity [19] (Figure S1). In this model, GABAergic neurons from the striatum project monosynaptically to the globus pallidus internus (GPi) and substantia nigra reticulata (SNr) in the direct pathway and polysynaptically to the GPi/SNr via the globus pallidus externus (GPe) and subthalamic nucleus (STN) in the indirect pathway. As the predominant output of the basal ganglia is inhibitory from the GPi/SnR to the thalamus, the direct pathway inhibits the GPi/SNr and releases thalamic and cortical neurons, whilst the indirect pathway excites the GPi/SNr and inhibits thalamic and cortical neurons. Hypokinetic movement disorders (such as Parkinson's disease) arise due to excess activity of the indirect pathway whereas hyperkinetic disorders (such as dystonia) arise due to excess activity of the direct pathway [20].

**Fig. 1:**
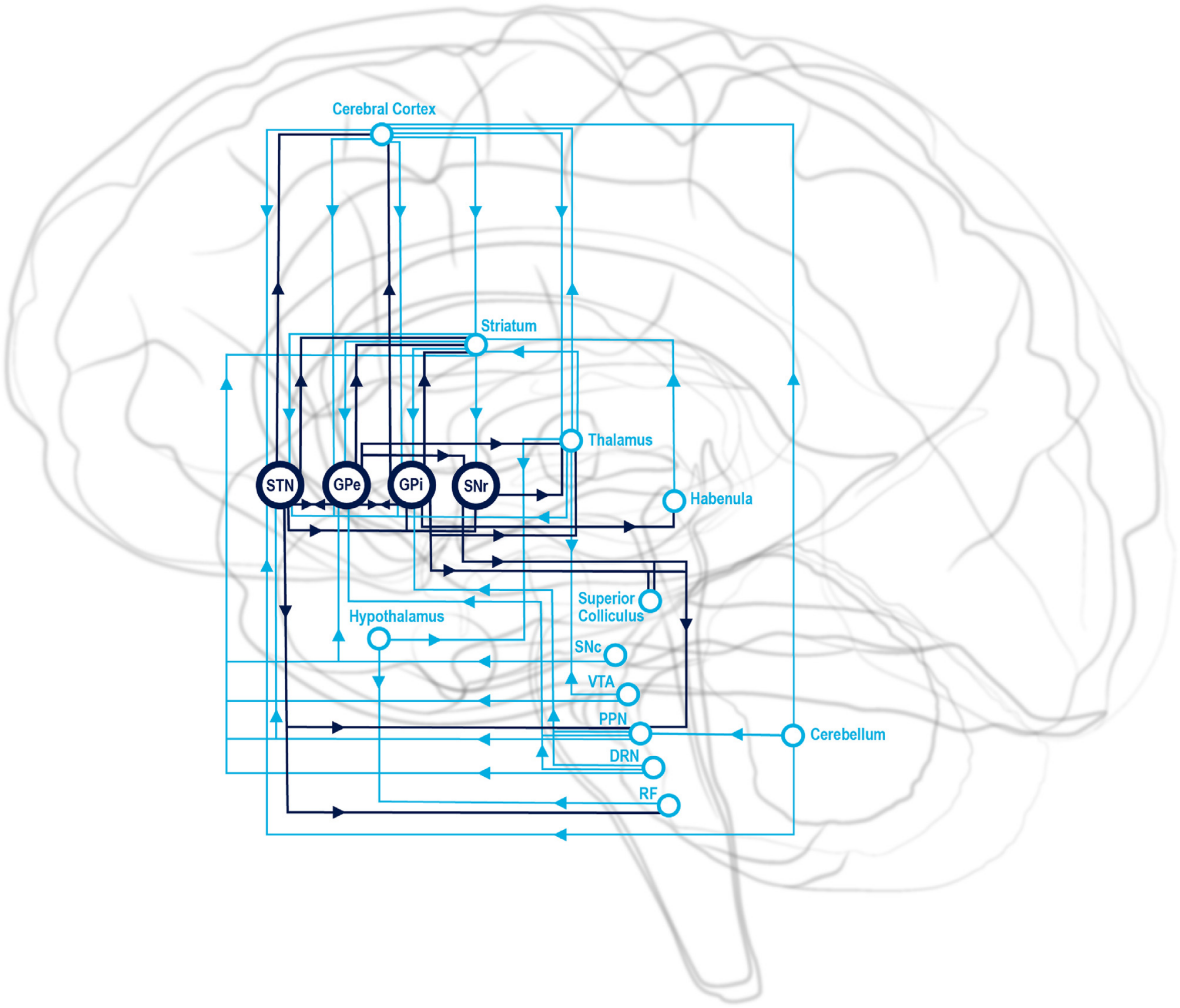
Relationships of the basal ganglia nuclei, with selected interactions between hypothalamus, thalamus, cerebellum and habenula. Abbreviations: DRN (dorsal raphe nucleus), GPe (globus pallidus externus, GPi (globus pallidus internus), PPN (pedunculopontine nucleus), RF (reticular formation), SNc (substantia nigra compacta), SNr (substantia nigra reticulata), STN (subthalamic nucleus), VTA (ventral tegmental area).

Whilst this model was useful for modelling the pathophysiology of movement disorders, it has been superseded by newer anatomical studies [21] and the conception of the brain as a dynamic organ rather than a static structure with input/output routines [22]. Recent studies have significantly expanded our understanding of the anatomical and functional connections of the basal ganglia. The striatum serves as the gateway and major integrating centre of the basal ganglia, receiving afferent input from the whole brain, around half of which consists of a topographical projection from the cerebral cortex. This information is channelled through the GPi, SNr, GPe and STN, with each nucleus having extensive connections with each other and variably to the cerebral cortex, thalamus, hypothalamus, limbic system, basal forebrain, brainstem nuclei and cerebellum (Supplemental Table S1 and S2, Fig. 1). Animal studies have formed the basis of models of basal ganglia function in humans, as the functional organisation of the basal ganglia is highly conserved and nearly identical in rodents and primates [21].

## Role of the basal ganglia in sleep

### Anatomical relations between the basal ganglia and structures regulating the sleep-wake cycle

The connections of the basal ganglia nuclei make it likely that they play an active, rather than passive, role in sleep. There are reciprocal connections between the basal ganglia and every part of the sleep-wake circuit: the cerebral cortex, brainstem nuclei, basal forebrain, thalamus, and hypothalamus (Supplemental Table S2 & Fig. 1). The ascending arousal system has been conceptualised in terms of ventral and dorsal streams based on early experiments [23]. However, it is now apparent that various components of the basal ganglia receive direct input from components of the ascending arousal system and may in fact be an integral part of it. There are other anatomical areas where boundaries between structures are indistinct such as the ventral striatum (nucleus accumbens and olfactory tubercle) and pallidum which merges with the basal forebrain and amygdala [24] and the subthalamic nucleus, the medial border of which merges with the lateral hypothalamus [16].

### Striatum and sleep

The rich innervation of the striatum from the cerebral cortex, thalamus, brainstem and other areas suggests that it may play an important role in sleep. Recordings from the striatum in rodents show that rhythmic changes accompany the sleep-wake cycle; slow wave bursts in NREM sleep and desynchronization in the waking and REM state [25]. A variety of animal studies have demonstrated changes to sleep patterns that accompany lesions in the striatum, including changes in time spent in waking and NREM sleep [26], reduced time spent in wakefulness [27], increased time spent in REM sleep [12] and reduction in NREM sleep with nucleus accumbens (NAc) core lesions [27].

**Fig. 2:**
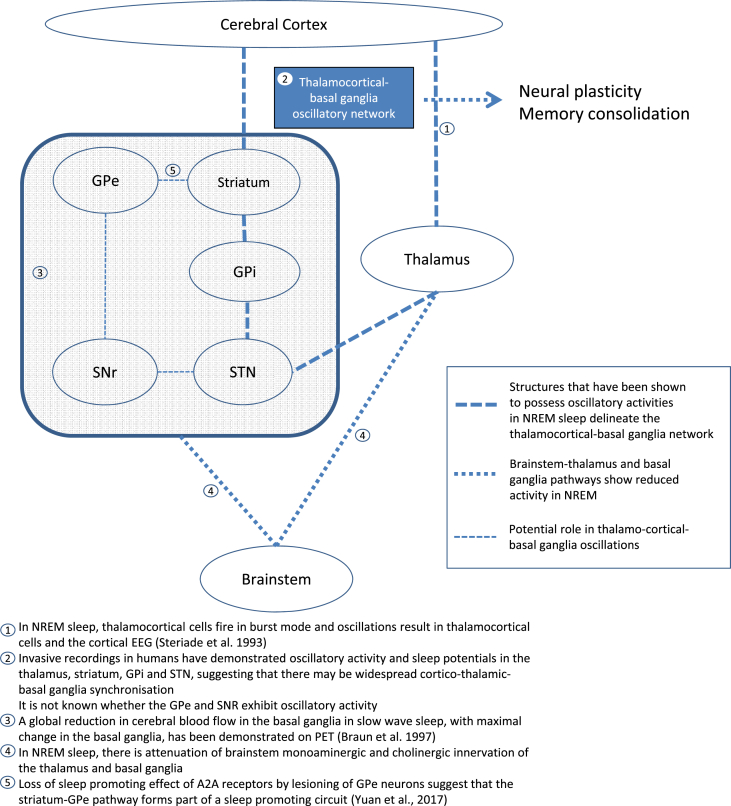
Role of the basal ganglia in sleep pathways.

Recent studies on adenosine receptors have also highlighted the role of the striatum in sleep. A variety of adenosine receptor subtypes are found throughout the body and in the brain, for example in the basal forebrain where adenosine A_1_ receptors are believed to promote sleep by inhibiting wake-promoting neurons [28]. Adenosine A_2A_ receptors are expressed on medium spiny neurons projecting to the GPe [29]. The A_2A_ receptor in the NAc shell has been found to mediate the arousal effect of caffeine [30], as well as chemogenetic and optogenetic stimulation of A_2A_ expressing neurons in the NAc core promoted sleep [31]. Lesions of GPe neurons associated with A_2A_ expressing striatopallidal neurons abolished its sleep-promoting effect, suggesting that this striatum-GPe pathway forms part of a sleep-promoting circuit mediated by A_2A_ receptors (see Fig. 2, *pathway 5*) [32].

Some studies suggest that dopaminergic effects on striatal neurons may also influence sleep. Rats deficient in dopamine afferents to the striatum were found to spend more time awake [33] and microdialysis in rodents found high extracellular fluid dopamine in the NAc during waking and low levels in NREM sleep [34]. Given that dopamine D_2_ receptors are co-expressed with A_2A_ receptors on striatopallidal neurons, it has been speculated that dopamine from the VTA may mediate cognitive-motivational influences on sleep, such as staying awake during a boring lecture [35]. Additional support of the role of the striatum in sleep is given by the high prevalence of sleep disorders in patients with Parkinson disease and Huntington's disease, both of which involve striatal dysfunction [36,37]. Both adenosine and dopamine act on multiple receptor subtypes throughout the striatum, rest of the basal ganglia and the body and may exert wake-sleep effects through different mechanisms [38]. It remains difficult to assign a sleep-wake role to a neurotransmitter alone, and it would be important for future work to address the spatial topography and receptor subtypes of the relevant neurons within the striatum and the efferent connections through the basal ganglia by which they may connect to known sleep and wake promoting centres [39].

The relationship between the striatum and REM sleep is of interest. In cats, acetylcholine increases in the caudate nucleus during REM compared to NREM and waking states [11]. In humans, PET and MRI studies show reductions in CBF in the striatum on going from waking to NREM sleep, and an increase in REM compared to NREM [40]. The striatum is one of the most densely innervated cholinergic structures of the brain. The aspiny interneurons account for the majority of this, but there is a proportion accounted for by projections from the brainstem and basal forebrain which has been demonstrated in humans [41]. The cholinergic innervation is part of the ascending arousal system and is active during waking and REM sleep [42]. Cholinergic innervation may be an important part of REM networks which allow the brain to draw on cognitive, affective and memory circuits via the basal ganglia that facilitate dreams and memory consolidation [43,44]. This function may be ‘off-line’ in NREM sleep, during which the striatum exhibits typical oscillatory activity [25].

### Pallidum and sleep

The function of the globus pallidus during the sleep-wake cycle may be reflected in the activity of the striatum, as the GABAergic input from medium spiny neurons forms a substantial part of its afferent input. Several studies in rodents have demonstrated changes in the sleep-wake pattern with lesioning or stimulation of the pallidum. Lesioning the GPe and GPi in rats led to decreased sleep, increased total wakefulness and fragmentation of sleep-wake behaviour including more sleep-wake transitions and shortened sleep bouts [27,33]. Optogenetic [33] and electrical [45] stimulation of the GPe in rats significantly increased nREM and REM sleep time. One could speculate that this could be due to reduced inhibitory output to the thalamus, but there is not enough evidence to support this and, in addition the lesions in the animals were incomplete. Of interest is that the firing pattern in the globus pallidus did not change significantly throughout the sleep-wake cycle (although the rate was higher in waking and REM than in slow wave sleep) [46], suggesting that, in contrast to the striatum, its functions may span across vigilance states and that its intrinsic functions may not be related to levels of arousal.

### STN and sleep

Recording from the STN in rodents and humans across the sleep-wake cycle shows that neurons change from random discharge in waking to a rhythmic bursting pattern in slow wave sleep, and increase its firing rate in REM sleep [46,47]. In humans, spindles and K-complexes are seen in stage 2 of NREM sleep and delta and theta activity in stage 3 [47]. Lesioning the STN in rats did not have an effect on sleep architecture [27]. Several studies have reported improvements in sleep following STN DBS [[48], [49], [50], [51]], but the variety of factors that may impact on sleep after deep brain stimulation render these findings difficult to interpret.

### Thalamo-basal ganglia connections and sleep

The thalamus is traditionally considered as a station that connects the basal ganglia outputs from the GPi and SNr to the cerebral cortex, but there are extensive thalamic afferent connections to the basal ganglia as well as efferent input to the thalamus from the GPe and STN. Thalamostriate projections arise from the midline and intralaminar nuclei and from the dorsomedial and VA/VL nuclei, and project to functionally discrete areas of the striatum [52]. The midline and medial intralaminar nuclei project to ventral (limbic) striatal areas whereas the more lateral intralaminar nuclei have connections with dorsolateral caudate and putamen [53]. The CM-PF projections also innervate the globus pallidus [1], substantia nigra and subthalamic nucleus. The thalamic nuclei provide direct feedback to the basal ganglia as well as to the cerebral cortex. The thalamocortical neurons and thalamic reticular nucleus have well established roles in the physiology of sleep [54]. Whether or not thalamocortical oscillations influence basal ganglia activity or whether there exist distinct thalamo-basal ganglia oscillations remains to be determined. The intralaminar and reticular nucleus of the thalamus receive afferent input from the major outflow of the basal ganglia through GPi, GPe and SNr, and project back to the striatum, GPi, GPe and STN as well as to the cerebral cortex, and receives ascending input from the monoaminergic and cholinergic ascending arousal systems. These anatomical relationships suggest that the thalamus plays a role beyond a relay station, but more studies are required to decipher the role of thalamo-basal ganglia interactions in sleep and wakefulness.

## Functional imaging studies of sleep that highlight the involvement of the basal ganglia

Functional imaging with PET and fMRI has increasingly been used to investigate the functional anatomy of sleep, often with concurrent EEG in a block design to identify imaging differences between waking, NREM and REM sleep [55]. However, few imaging studies have considered the role of the basal ganglia in *a priori* hypotheses [56]. Most recent sleep imaging studies do not mention the basal ganglia other than comment on their involvement in passing [57].

The ascending arousal systems were shown active during REM but not during slow wave sleep [15]. Similar findings were found in subsequent PET and MRI studies [15,40,[58], [59], [60]]. Reduced connectivity of the inferior parietal lobule with the caudate in light sleep was found in a fMRI study [57] but the significance of this is difficult to interpret.

In an important study highlighting the importance of the basal ganglia in sleep, Braun et al., 1997 performed a PET study in healthy sleep-deprived subjects and compared cerebral blood flow in pre-sleep waking, slow wave sleep, REM sleep and post-sleep waking conditions [15]. They found that slow wave sleep was associated with a global reduction in CBF compared to waking and REM [15]. The most statistically robust changes occurred in the basal ganglia – the posterior putamen and caudate nucleus. Other decreases were seen in the pontine tegmentum, basal forebrain, cerebellar hemispheres, thalamus, paralimbic areas, mesial temporal structures and higher association cortical areas, suggesting diminished activity overall. There was a significant increase from slow wave sleep to REM in the putamen and caudate nucleus, and also basal forebrain, cerebellum, limbic areas, insula, the sensorimotor, visual and auditory association cortices, and all regions of the brainstem and thalamus, suggesting that there is a generalised activation in REM, and in some areas such as the pontine tegmentum, midbrain, basal forebrain, cerebellar vermis, and caudate nucleus, even more so than in the waking state.

## Reports of patients undergoing interventions in the basal ganglia

### Intracranial recording during sleep from deep brain structures from DBS electrodes in humans

Deep brain stimulation (DBS) is increasingly used to treat a variety of neurological disorders including Parkinson's disease, dystonia, tremor, pain, and epilepsy [18]. Several studies have utilised LFP recordings from externalised DBS electrodes with polysomnography to investigate the role of subcortical structures in sleep in humans [47,56,[61], [62], [63], [64], [65], [66], [67], [68], [69]].

In one of the earliest reports, Moiseeva et al. (1969) [61] described recordings during sleep from 11 patients, with electrodes in various locations including the subthalamic nucleus, pallidum, putamen, caudate nucleus, substantia nigra, ventrolateral and posterior thalamus and hypothalamus [61]. They reported that the electrical patterns corresponding to the various stages of sleep develop in different ways in different structures. They also reported changes in the firing rate in the subthalamic nucleus, ventral thalamic area and hippocampus during the development of sleep, and concluded that these structures play an essential role in sleep [61].

Spectral analysis of the subcortical LFP in sleep has been reported in four studies, with electrodes in the STN in patients with Parkinson disease [47,63], GPi in patients with dystonia [56] and VIM in patients with myoclonic tremor and myoclonic epilepsy [62]. STN recordings were found to resemble those of the scalp, with higher frequencies dominating the power spectra in awake and REM, and slower frequencies predominating in NREM sleep [47,63]. These findings support the involvement of the STN in sleep, but their significance is difficult to interpret. The STN LFPs were analysed in bipolar derivations across the DBS electrode contacts (0–1, 1–2 and 2–3) in both studies and the scalp EEG was referenced to bipolar (F3–C3, P3–O1, F4–C4, P4–O2) in the Thompson et al. (2017) [63] study and the common average in the Urrestarazu et al. (2009) [47] study. Whilst the observation that similar frequencies dominate both the scalp and subcortical LFPs during sleep may be in support of the action of a thalamocortical mechanism regulating the sleep-wake cycle [70], how such mechanisms result in simultaneous localised (STN) and distributed (scalp) oscillations and how they relate to the activity of specific anatomical networks is unknown. Interestingly, no association was found between GPi LFP spectra and that of the scalp [56]. This may indicate an element of functional segregation, although further studies are required to support such hypotheses. Kempf et al. [62]studied gamma activity in VIM across sleep stages in 2 subjects, and found that ∼70 Hz gamma activity (‘finely tuned gamma’) was present in awake but absent in NREM sleep, and recurred intermittently in REM [62]. They concluded that finely tuned gamma activity may be an indicator of activity of the ascending arousal system [62]. This hypothesis also requires further empirical evidence to support it.

Vertex waves, K-complexes and sleep spindles have been recorded during sleep from various subcortical structures (Table 1). These findings raise questions about the role of the subcortical structures in sleep, and of the origin and function of sleep potentials.

**Table 1:** Sleep potential recorded from subcortical structures in humans.

Study	Patients	Electrode Placement	Monopolar or bipolar	Subcortical sleep rhythm	Notes
Urrestarazu et al. (2009) [47]	N = 10, PD	STN	Bipolar subcortical, EEG common average	K-complex, Spindles	Some spindles occurred simultaneously in Cz. Subcortical spindles not recorded in all patients. Half of patients recorded during daytime naps rather than nocturnal sleep
Velasco et al. (2002) [67]	N = 5, Lennox-Gastaut Syndrome	CM	Monopolar (ipsilateral earlobes)	K-complex, Vertex waves, Spindles	Vertex waves occurred in scalp first, spindles occurred in thalamus first
Moiseeva et al. (1969) [61]	N = 11, diagnosis not specified	Globus pallidus, putamen, hypothalamus, ventrolateral & posterior thalamic nucleus, hypothalamus, STN, substantia nigra)	Not documented	Spindles	Abstract only
Salih et al. (2009) [56]	N = 7, dystonia	GPi	Bipolar	K-complexes Spindles	Subcortical potentials occurred in association with scalp.
Tsai et al. (2010) [68]	N = 3, epilepsy	Anterior nucleus	Bipolar	Spindles	Spindles occurred at the same time in anterior nucleus and scalp. K complexes only seen on scalp potentials.
Wennberg & Lozano (2003) [65]	N = 7, epilepsy, PD	Centromedian nucleus, anterior nucleus, STN	Monopolar to Cz, Pz or sphenoidal electrode	K-complex Vertex waves Spindles	Sleep potentials occurred simultaneously and in opposite polarities

*Abbreviations*: CM (centromedian nucleus), GPi (globus pallidus internus), PD (Parkinson's disease), STN (subthalamic nucleus).

The appearance of sleep potentials at distant locations occurring in close temporal association indicates that the same fluctuation in potential difference occurs between the pairs of recording electrodes in the respective locations. This could be explained by neural propagation, volume conduction or activity at a common reference electrode. That this occurs due to volume conduction is less likely given that on some occasions sleep spindles appeared independently, either in the scalp or CM nucleus [67]. In the study by Velasco (2002) [67], scalp vertex waves significantly preceded the thalamic counterpart (by 42 ± 5 ms) and CM spindles preceded their scalp counterpart (by 512 ± 50 ms), leading the authors to suggest that vertex waves originate in the scalp whilst sleep spindles originate in the thalamus, whereas Wennberg and Lozano (2002) [71] reported that sleep potentials appeared simultaneously in the scalp and subcortical structures but with opposite polarity, leading them to conclude that subcortical sleep potentials are volume conducted from the cerebral cortex [71]. As the recordings performed by both Velasco et al. [67] and Wennberg and Lozano [71] utilised monopolar references, it could be argued that activity at the common reference electrode could have generated the waves in the two distant locations, although this would not explain the temporal asynchrony.

A more potent finding that weighs against reference electrode or volume conduction effects is that subcortical sleep potentials have also been recorded from bipolar contacts in the STN [47] and thalamic anterior nucleus [68]. Urrestarazu et al. (2009) [47] reported the presence of spindles and K complexes in bipolar STN LFPs during stage 2 sleep, although only in 6 out of 10 patients [47]. Some sleep spindles were also seen at the same time in Cz. Tsai et al. (2010) [68] found that sleep spindles occurred simultaneously on the scalp and in the thalamic anterior nucleus on bipolar recordings, but K complexes were only seen on the scalp [68]. Fluctuations in local field potentials broadly reflect fluctuations in intracellular activity [72,73]. Whilst changes in potentials seen on a bipolar montage are often attributed to local changes in neuronal activity [74], this may not always be the case [75] and these findings raise the question of why monopolar and bipolar recordings should both generate sleep potentials of similar morphology in close temporal association in the scalp and subcortical structures.

The K complex is considered to arise in the cortex of the cerebral hemispheres rather than the subcortical structures [76,77]. A number of findings support this hypothesis, including the widespread spatial distribution of the K-complex over the cerebral hemispheres on intracranial recordings [74,76,78], the electrographic and physiological association of the K complex with the cortically-generated slow oscillation [77], the polarity reversal of the K-complex when cortical and subcortical recordings are compared [79] and the fact that stimulation of cortical areas induce K-complex-like waveforms [76]. It is difficult to reconcile these findings with the observation of K-complexes on bipolar STN [47] and GPi [56] recordings.

These studies indicate that sleep oscillations and potentials occur and are similar on the scalp and a variety of subcortical structures, and that sleep potentials may be associated or dissociated from scalp potentials but there is insufficient data in these small number of studies to fully explain these findings. One potential explanation for subcortical spindles and K-complexes is neuronal synchronization [70]. Thalamic spindles are synchronized across the length of an electrode, and this is dependent on feedback from the cortex [80]. If such a mechanism is responsible, the findings demonstrating sleep potentials in the basal ganglia would greatly extend the scope of the role of the basal ganglia in sleep. It has been hypothesized that the slow (<1 Hz) oscillation may group spindles and delta waves [54,70]. If this is the case, one would expect to see slow oscillations in the basal ganglia, but this has not been well described [68].

Confounding issues in these studies are the diversity of targets and recording methodologies, the reliability of data, particularly with older studies in which the accuracy of measurement may be uncertain [71], the limitations on what can be concluded from LFP measurements which necessarily include volume conducted effects, and the fact that these studies are all performed on patients with underlying medical conditions on medication. Whilst the latter factor is unavoidable in intracranial recording studies in humans, it would be helpful to have a strategic approach to intracranial sleep studies within which results can be compares across studies.

On some level, the neural processes that give rise to the various oscillations and potentials must cause the profound behavioural and cognitive changes that accompany the various stages of sleep. Whether or not the oscillations and patterns themselves can be traced back to neuroanatomical pathways and/or whether their corresponding physiological, cognitive and behavioural correlates can be identified remains a fertile field for further investigation. It is tempting to use EEG waveforms (whether scalp or intracranial) as a bridge to connect neurophysiology and behaviour but its limitations must always be borne in mind. Could concepts such as ‘the location where sleep potentials are generated’ and discourse such as ‘the propagation of sleep potentials’ be misguided? Is there a site of origin? Are waveforms truly propagated or does it appear so by the way in which the EEG trace is constructed, and what relevance does it have to underlying neural activity? Important questions remain, but there is probably enough evidence from these intracranial studies in humans to suggest that it is premature to discount the role of the basal ganglia in sleep.

### Sleep EEG in patients who have had DBS electrodes internalized: the effect of subcortical stimulation on sleep

The study of sleep behaviour and EEG sleep architecture in patients after their DBS systems have been internalised also shed some light on how modulation of basal ganglia activity affect sleep and its rhythms. Most studies involve patients with Parkinson's disease who have undergone STN implantation and examined sleep behaviour and architecture before and after DBS [48,[81], [82], [83], [84], [85]] or after DBS in DBS ‘on’ and ‘off’ conditions [86] (Table 2).

**Table 2:** Sleep characteristics before and after DBS.

Study	DBS target	Sleep measure	Effect
Iranzo et al. (2002) [49]	STN	PSG, clinical interview, PSQI	PSQI showed significant improvement. PSG showed more continuous sleep.
Hjort et al. (2004) [116]	STN	Parkinson Disease Sleep Scale	Improves sleep quality mainly due to motor improvement
Cicolin et al. (2004) [48]	STN	PSG	Improvement in sleep architecture but not PLM or RBD
Baumann-Vogel et al. (2017) [82]	STN	PSG, ESS, Zurich sleep questionnaire	DBS reduced sleepiness and improved some PSG parameters but did not normalise sleep.
Tolleson et al. (2016) [84]	GPi	PSG	No effect on sleep parameters
Lim et al. (2009) [86]	PPN	PSG	DBS ‘on’ increased REM time compared to DBS ‘off’
Arnulf et al. (2010) [85]	PPN	Behavioural	Low frequency stimulation led to arousal whereas high frequency stimulation led to REM sleep

*Abbreviations*: ESS (Epworth Sleepiness Scale), GPi (globus pallidus internus), PLM (periodic limb movements), PPN (pedunculopontine nucleus), PSG (polysomnography), PSQI (Pittsburgh Sleep Quality Index), RBD (REM behaviour disorder), STN (subthalamic nucleus).

These studies suggest that for STN stimulation there is a clear effect towards improvement of sleep on both subjective and objective measures. It is, however, difficult to infer the mechanism by which STN stimulation leads to improved sleep in these situations because of the multiple aetiologies of sleep disorders in Parkinson's disease [36] but also because the mechanism of action of DBS remains poorly understood [87]. Interestingly, improvement in sleep was not seen after GPi DBS for Parkinson's disease [84], although more studies are required to draw implications based on this observation. Patients who had PPN DBS for Parkinson's disease [85] or progressive supranuclear palsy [86] have shown an improvement in sleep with low stimulation and induction of REM at high frequency stimulation, suggesting that stimulation directly affects sleep regulating pathways and verifies findings from anatomical studies suggesting that this region plays a crucial role in sleep regulation. Due to the variability of PPN anatomy and current spread it is impossible to specify which pathways are being activated in such situations. Therefore the most that can be concluded is that STN DBS leads to an improvement in sleep. The study protocols adopted to date, that of performing PSG before and after or during on and off DBS, are insufficient to study mechanisms of action. This will require a careful study whereby the parameters of stimulation are varied and the effects of this observed on behaviour and sleep architecture.

### Sleep disorders in patients with disease affecting the basal ganglia

Sleep disorders are highly prevalent in psychiatric and neurologic diseases which affect the basal ganglia. Up to 98% of patients with Parkinson disease [88] and 88% with Huntington's disease [89] report nocturnal sleep problems. In most cases the basal ganglia pathology is part of a wider process affecting other parts of the nervous system and it is difficult to implicate the basal ganglia as the cause of the sleep disturbance (Table 3). The cause of the sleep disturbance is often multifactorial and may include the persistence of motor symptoms at night, pain or discomfort, depression and anxiety, medical problems such as arthritis or nocturia, and medication side-effects. The pathophysiology of disease may also directly affect sleep-wake regulating centres. For example, Huntington's disease (HD) is characterised by striatal degeneration but also involves hypothalamic degeneration, and altered expression of circadian clock genes in the hypothalamus was found in a mouse model of this condition [37]. Parkinson's disease is associated with the widest variety of sleep disorders but this may reflect the prevalence of the condition and a high number of studies in this patient group in comparison with others.

**Table 3:** Sleep disturbance in disorders affecting the basal ganglia.

Disease	Basal ganglia involvement	Associated sleep disorders
Parkinson disease [36,88]	Nigrostriatal degeneration	Nocturnal awakening
		Sleep fragmentation
		Periodic limb movements/RLS
		REM behaviour disorder (RBD)
		Obstructive sleep apnoea
		Excessive daytime sleepiness
Huntington's disease [117]	Striatal degeneration	Nocturnal awakening
		Increased nocturnal movements
		RWA, PLM, RBD reported in a minority of patients
Progressive supranuclear palsy [118]	Neuronal loss and gliosis	Nocturnal awakening
		RBD reported in a minority of patients
Wilson's disease [119]	Copper deposition	Excessive daytime sleepiness
		Poor nocturnal sleep
		Cataplexy-like episodes
Pantothenate kinase-associated neurodegeneration (PKAN) [120]	Iron deposition	Reduced total sleep time

*Abbreviations*: PLM (periodic limb movements), RBD (REM behaviour disorder), RLS (restless legs syndrome), RWA (REM without atonia).

Study of sleep disorders in basal ganglia disease may reveal important connections between the functional anatomy of sleep and the pathophysiology of disease. A good example is the case of REM behaviour disorder (RBD), which involves loss of muscle atonia in REM sleep and enactment of dreams. A careful study of the association of RBD with various degenerative condition affecting the basal ganglia and brainstem has highlighted the sublaterodorsal nucleus (SLD) as an important mediator of this condition [90]. The SLD has descending connections with spinal motor neurons and is an important part of the REM switch [91]. The onset of RBD decades before clinical presentation of Parkinson's disease (PD) and other synucleinopathies suggests that PD begins outside the basal ganglia [90,92]. Stimulation of the SnR attenuates REM atonia, which suggests that the basal ganglia may have a role in regulating REM, REM atonia and therefore may also be involved in RBD [93]. Higher prevalence of periodic limb movement disorder in RBD patients has been argued to reflect their shared basal ganglia etiology [94,95]. Moreover, some authors have even hypothesised that earlier onset of RBD or PD in a course of neurodegeneration may depend on whether the dorsal or ventral part of the brainstem are initially involved [96]. For instance, nigro-caudate dopaminergic deafferentation has been argued as a principal biomarker of RBD [96], with several recent studies demonstrating an early involvement of caudate and ventral striatum in PD patients with overall worse outcomes, and with an increased risk of developing sleep issues with debilitating cognitive and mood problems [96,97]. In keeping, neuroimaging studies of PD have consistently demonstrated uneven dopaminergic deficit within the striatum, with more severe involvement of the posterior putamen and a relative sparing of the head of caudate nucleus [97,98]. Perhaps in similar vein, sleep disturbances and striking mood pathology in HD [99] have been linked to early neuronal loss in the medial caudate [100]. While there is no homogenous pattern of sleep disorders in patients with HD, insomnia, increased sleep onset latency, decrease in total sleep time, frequent nocturnal awakenings, REM sleep disorders, increased motor activity during sleep, decreased sleep efficiency and excessive daytime sleepiness have all been reported (for in-depth review see [101]). Reduced N3 and REM stage and an increased sleep spindle density have also been observed in HD patients [101].

The relationship between basal ganglia and psychiatric disorders has traditionally been either ignored, or at best considered subservient to historically widely recognised links with motor and other neurologic disorders. However, the awareness for the pivotal role of basal ganglia neurocircuitry in the pathology of major psychiatric disorders has significantly improved within the last few decades (as comprehensively reviewed in [102,103]). In keeping with this, DBS of basal ganglia has also been increasingly recognised as a promising treatment of severe and refractory psychiatric disorders. For example, in patients with obsessive–compulsive disorder (OCD), which is characterized by chronic intrusive thoughts or impulses (obsessions) and ritualistic and repetitive actions (compulsions), DBS treatment of two main regions, the striatal region (including the caudate nucleus, NAc, anterior limb of the internal capsule, and the ventral capsule) and the STN, has shown promising results in reducing OCD symptoms, and in normalising connectivity within corticobasal ganglia and thalamocortical circuits (for review see [102]). Sleep pathology has been described in this patient population, with evidence for reduced total sleep time and sleep efficiency, a delayed sleep onset and offset and an increased prevalence of delayed sleep phase disorder [104]. Comorbid sleep issues have also been recognised as an important moderating factor in the prognosis and severity of OCD [104].

Similar to OCD patients, dysfunction of basal ganglia has been suggested as central to pathology underlying development of addiction and substance abuse disorders [102]. Hence, given the grave impact and socio-economic consequences that such disorders pose to the individual and the society, the relationship between substance abuse and sleep has emerged as an area of great interest for clinicians and researchers (see [105]. Patients with substance abuse have been shown five to ten times more likely to have sleep issues, and presence of this comorbidity has significant impact on severity of addiction, remission and the related relapse prospects, as well as prevalence of affective symptomatology and depression [105]. Analogously to demonstrated links between ventral striatum, sleep and aberrant behaviors in RBD, PD, HD and OCD, hypoactivity (or hyperactivity) of the ventral striatum (VS) has similarly been reported in substance users [106]. Of note, ventral striatum has long been recognised as a central region of the reward circuit, and its role in drug addiction and in the persistence of maladaptive behaviors such as compulsion to seek the drug, has long been been hypothesised [106]. More recently, a low-frequency DBS of the dorsal VS has been shown useful in treatment of refractory opioid addiction [106], whilst, in past, high-frequency DBS of the VS has also been shown effective in reducing symptoms of addiction to alcohol, nicotine, and heroin [106]. Within that context, of note are findings of a recent study that suggested that high activity of VS may buffer against the experience of depressive symptoms, associated with sleep disturbances [107]. The aberrant activity of VS, with or without associated anhedonia and apathy, has been implicated in several other psychiatric disorders, such are for example attention deficit hyperactivity disorder (ADHD), characterized by symptoms of age-inappropriate inattention, hyperactivity and impulsivity [108], and schizophrenia [109]. In recent comprehensive assessment of sleep disorders and their correlates in patients with early psychosis, sleep disorders were significantly associated with increased psychotic experiences, depression, anxiety, fatigue, and lower quality of life [110]. The authors highlighted strong links between sleep disorders and psychosis, and suggested that their findings suggest that comorbid sleep pathology may have wide-ranging negative effects, and that it hence merits routine assessment and treatment in psychiatric practice [110].

In conclusion, while the translational neuroscience of mechanistic corelates that may bind sleep disorders and neuropsychiatric disorders with dysfunction of nigrostriatal pathways is far from clear, some speculative theoretical patterns have emerged. For instance, in genetically predisposed individuals and or under extreme conditions, such as psychosis, where the central nervous system pathology may lead to excessive dopamine striatal transients [111], these may lend to irrelevant external or internal stimuli being marked as of overvalued/delusional ‘significance’, due to their temporal association of the stimuli with striatal signalling [109]. Following that concept, arguably, the aberrant association within dorsal regions of the striatum may be tied to signalling threat-related information, and possibly lead to delusions or indeed to nightmares (if they occur during REM), which are both frequently persecutory in nature [110]. Impaired reality assessment may further increase vigilance in any affected individual and lead to decreased sleep efficiency, which may in turn further affect striatal dopaminergic functioning [112]. Comparably to this theoretical construct, the association with the ventral striatal regions may result in the hypervigilance, insomnia, and unexpected or aberrant reward associations leading to addictions, obsessions and attention deficits [104,113].

## Conclusions

The reciprocal connections between the basal ganglia and the sleep-wake circuitry, and the experimental findings reviewed in this paper point to an important role of the basal ganglia in sleep (Fig. 2). A major challenge is to define the role of specific neuronal populations and their relationship to cellular oscillations and scalp EEG rhythms. The role of the basal ganglia in processing sensorimotor, cognitive and affective information is well established, and it would be reasonable to hypothesize that these processes may continue during sleep, particularly in REM sleep, which draws on these processes in the emergence of conscious experience in dreaming, a hypothesis which finds some experimental support [43]. There is also evidence to suggest that the striatum, GPi and STN adopt rhythmic oscillations during NREM sleep. More studies are required to confirm these findings and their relationship to thalamocortical oscillations, including the prospect for a thalamocortical-basal ganglia oscillatory network for neural plasticity and memory consolidation. On the other hand, patients in whom parts of the basal ganglia are absent [114] indicate that sleep can be sustained without those structures. This may suggest a facilitating, rather than constitutive role of the basal ganglia in sleep. Sleep is accompanied by profound changes in the motor system, which may be facilitated by the basal ganglia. The observation that many movement disorders which are presumably of basal ganglia can disappear during sleep [115] give support to this, and may signal an untapped resource for neuromodulation. The precise role of the basal ganglia in the different aspects of sleep, including the homeostatic and cognitive mechanisms involved in going to sleep, the regulation of stages of sleep, the effect of sleep on muscle tone, memory effects, sleep disorders and dreaming and the regulation of consciousness, all merit further study. This would hopefully bring us toward a better understanding of why living organisms have been designed to sleep.

**Practice points**

The modulatory role for the basal ganglia in sleep regulation is suggested by:

1) emerging evidence that the striatum, GPi and STN structures adopt rhythmic oscillations during NREM sleep;
2) their active role in processing sensorimotor, cognitive and affective information during wake and sleep states;
3) emerging molecular studies on the sleep and wake promoting effects of specific neuronal populations projecting to and from the basal ganglia.

**Research agenda**

Future studies should decipher the precise role of the basal ganglia in the different aspects of sleep:

1) including the homeostatic and cognitive mechanisms involved in going to sleep;
2) the regulation of stages of sleep;
3) the effect of sleep on muscle tone;
4) memory effects and consciousness;
5) and last but not least, in sleep disorders.

## Conflicts of interest

All authors were involved in reviewing and drafting of the manuscript. The authors declare that the research was conducted in the absence of any commercial or financial relationships that could be construed as a potential conflict of interest.
