# Supplementary material for: The subcortical belly of sleep: New possibilities in neuromodulation of basal ganglia?
Source: Sleep Med Rev. 2020 Aug;52:101317. doi: 10.1016/j.smrv.2020.101317 (PMC7679363; doi:10.1016/j.smrv.2020.101317)
Supplement: Multimedia component 1 [file mmc1.docx]

**Figure S1.** Basal Ganglia Circuits: Direct and Indirect Pathways.

**
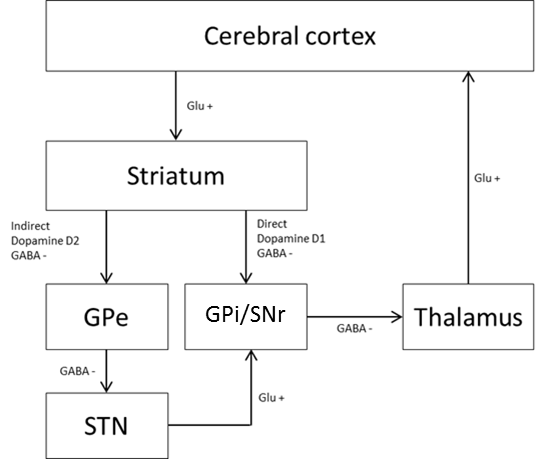
**

**Table S1: Wake, sleep and REM promoting mechanisms in the brain.**

| **Anatomical Region** | **Main Neurotransmitters** | **Connections (human and rodent studies, afferent and efferent, see references for details)** | **Description of findings suggesting involvement in sleep** |
| --- | --- | --- | --- |
| **Predominantly Wake Promoting Areas** |  |  |  |
| **Brainstem** |  |  |  |
| Dorsal and median raphe nuclei | **Serotonin**  Dopamine  GABA  Glutamate | The dorsal raphe nucleus in primates contains the largest number of 5-HT neurons in the brain. Studies in rats, cats and humans show that these neurons project to all basal ganglia nuclei (1-3) as well as to the thalamus, hypothalamus, basal forebrain, limbic system, brainstem and cerebral cortex (4-6). | Firing of serotonergic neurons in dorsal raphe is highest in waking, lower in NREM and almost absent in REM, and serotonin levels in brain are higher in waking then sleep and REM (7, 8). However, serotonin receptor subtypes may have opposite effects on wake and sleep, selective serotonin uptake inhibitors have variable effects on wake and sleep, and some serotonergic dorsal raphe neurons fire during sleep (7). Lesioning the raphe nuclei had sleep promoting effects, this may be due to the effect of serotonin-induced hypothermia on sleep (9). Dorsal raphe dopamine neurons promote waking (10). |
| Locus coeruleus (LC) | Noradrenaline | Noradrenergic neurons from the LC innervate the entire CNS including basal ganglia. The end-organ effects are modulated by differences in peptide expression and receptors (11). | LC neurons fire steadily during awake, less during NREM and virtually silent during REM sleep (12, 13). Changes in LC activity precede EEG transitions from wake to NREM and NREM to waking (14). Optogenetic stimulation of LC causes sleep to wake transitions (15). However, lesions of LC do not produce consistent changes in EEG or behavioural arousal (16) and genetic ablation of the NE precursor dopamine decarboxylase does not affect sleep-wake states (17). |
| Ventral tegmental area (VTA) | **Dopamine**  GABA  Glutamate | Cerebral cortex, basal forebrain, hypothalamus, basal ganglia, limbic system, brainstem (18). | Activity of VTA dopamine neurons higher in waking and REM compared to NREM, inhibition of their activity increases sleep characteristics (19). |
| Pedunculopontine-tegmental nucleus (PPT) / laterodorsal nucleus (LDT) | **Acetylcholine**  **GABA**  **Glutamate** | The PPT contains cholinergic, GABAergic and glutamatergic neurons which project to the striatum, globus pallidus, STN, SNc, thalamus, hypothalamus, basal forebrain, pontine and medullary reticular formation, spinal cord, cerebellum and cerebral cortex. Major afferents to the PPT originate in the basal ganglia with projections from the GPi, STN and SnR. It also receives input from the orexin neurons of the hypothalamus, histaminergic neurons from the TMN, serotonergic input from the dorsal raphe, adrenergic input from the LC, and cholinergic input from the LDT and contralateral PPT (1, 20-23). | Cholinergic and some GABAergic and glutamatergic neurons in the PPT/LDT fire maximally during waking and REM. Some GABAergic and glutamatergic neurons only fired in REM. Some glutamatergic neurons were active in waking only (24). Chemogenetic activation of PPT glutamatergic neurons increased waking time, cholinergic neurons had no effect on waking/sleep time but reduced slow waves in NREM sleep, GABAergic neurons slightly reduced REM sleep (23). Optogenetic stimulation of cholinergic PPN and LDT neurons induced REM from NREM (25). Lesioning the PPT does not have substantial effects on sleep-wake architecture (26). |
| Parabrachial nucleus (PB) | **Glutamate**  Dopamine | Basal forebrain, intralaminar thalamus, lateral hypothalamus, amygdala, dorsolateral and medial prefrontal and insular cortex, VLPO | Lesions incorporating the parabrachial nucleus in rats cause coma (27). Chemogenetic activation of PB-extra thalamic (but not thalamic pathway) leads to increase in wakefulness (28) |
| **Ventral Stream of ARAS** |  |  |  |
| Basal Forebrain  (septal-diagnonal complex, medial part of globus pallidus, magnocellular preoptic nucleus, substantia inominata) | **Acetylcholine**  **GABA**  **Glutamate** | The basal forebrain receives afferents from a large area of the brainstem tegmentum including ventral tegmental area, substantia nigra, retrorubal field, raphe nuclei, reticular formation, PPN, LDT, PB and LC. Efferent fibres go to the amygdala, hippocampus, olfactory bulb, cerebral cortex (29, 30) | Basal forebrain neurons are active in waking and REM but not in NREM sleep (Lee et al. 2005). Complete BF lesion with OX-SAP leads to coma-like state and flat EEG, reduced Fos in cerebral cortex (but high Fos in brainstem, thalamus, hypothalamus) selective (cholinergic or non-cholinergic) lesion does not have this effect (27). Chemogenetic activation of BF GABAergic neurons facilitates wakefulness (31). Cholinergic, glutamatergic and parvalbumin (PV)-positive GABAergic neurons were more active during wake and REM whereas somatostatin (SOM)-positive GABAergic neurons were active during NREM. Optogenetic activation of cholinergic and glutamatergic caused transition from NREM to wakefulness and desynchronization of the EEG. PV+ GABAergic neurons promoted waking and SOM+ GABAergic activation promoted NREM (Xu et al.2015). |
| Tuberomamillary nucleus | **Histamine**  GABA | Cerebral cortex, thalamus, hypothalamus, basal forebrain, septum, olfactory bulb, amygdala, hippocampus, basal ganglia, brainstem, spinal cord (32). | Histaminergic neurons are active during waking states and silent during NREM and REM sleep (33). Optogenetic silencing of TMN histaminergic neurons promotes NREM sleep (34). |
| Preoptic hypothalamus | **GABA** | Hypothalamic nuclei, brainstem nuclei (dorsal raphe, LC, ventrolateral medulla, parabrachial nucleus), TMN, amygdala, cerebral cortex, claustrum (Chou et al., 2002). | Non-selective activation of preoptic GABA and glutamatergic neurons caused increase in waking (Chung et al., 2017). |
| Lateral hypothalamus | **Orexin** | Cerebral cortex, basal forebrain, intralaminar and relay nuclei of the thalamus, basal ganglia, amygdala, many components of the ascending arousal system including the LC, dorsal raphe, parabrachial nucleus (35, 36). | Orexin deficiency leads to narcolepsy (37). Orexin neurons fire during waking and virtually cease to fire in NREM and REM sleep (36). Optogenetic stimulation of orexin neurons has a wake promoting effect (38) whereas silencing led to induction of NREM sleep (39). |
| **Dorsal Stream of ARAS** |  |  |  |
| **Thalamus (midline, intralaminar and reticular thalamic nuclei)** | Glutamate  GABA | Cerebral cortex, basal ganglia, amygdala, hippocampus, cerebellum, brainstem. | Stimulation of thalamic areas including the intralaminar nuclei leads to cortical recruiting response (40). Tracing studies show pathways from brainstem nuclei and reticular formation to thalamic midline and intralaminar nuclei (41). Thalamic reticular cells generate spindles (42). Thalamic destruction eliminates spindles but does not inhibit cortical activation by brainstem stimulation in rats (27) and cats (43). Chemogenetic activaton of glutamatergic thalamocortical neurons had no effect on sleep-wake quantity, consolidation or sleep latency (31). |
| **REM regulating areas** |  |  |  |
| Mesopontine tegmentum (ventrolateral periaqueductal grey and lateral pontine tegmentum)  Sublaterodorsal nucleus (SLD) [subcoeruleus / peri-locus coeruleus alpha in cats]  Precoeruleus region  (see also PPT/LDT) | GABA  Glutamate | Ventrolateral and lateral hypothalamus, septum, locus coeruleus, dorsal raphe nucleus, PPT, medial pontine and medullary reticular formation and spinal cord. | Selective lesions of the ventrolateral periaqueductal grey or lateral pontine tegmentum doubled the amount of REM sleep in rodents, lesions of the SLD produce reductions in REM and loss of atonia (44). Optogenetic stimulation of ventrolateral periaqueductal grey GABAergic neurons promoted REM - these neurons fired most during REM, least during NREM and variable rates during waking (45). |
| **Predominantly Sleep Promoting Areas** |  |  |  |
| Lateral hypothalamus | **Melanin concentrating hormone (MCH)** | Medial septum, hippocampus, amygdala, basal forebrain, thalamus, hypothalamus, caudate, putamen, globus pallidus, periaqueductal grey, SNc, VTA, dorsal and median raphe nuclei, PPT/LDT, LC, pontine reticular formation (46). | MCH neurons are silent during waking, increase firing during NREM and fire more during REM (47). Optogenetic (48, 49) and chemogenetic (50) activation of MCH neurons promotes REM. Silencing of MCH neurons do not have substantial effects on sleep-wake (48, 49) |
| Pre-optic hypothalamus | **GABA**  Glutamate | TMN, dorsal raphe nucleus, LC, lateral hypothalamus, parabrachial nucleus), amygdala, cerebral cortex (51). | Optogenetic activation of preoptic GABAergic neurons projecting to the TMN increased NREM and REM sleep, inactivating them caused increased wakefulness and decreased NREM and REM sleep (52) . See (53) for a review. |
| Abbreviations: 5-HT (5-hydroxytryptamine), CNS (central nervous system), GABA (Gamma amino butyric acid), GPi (globus pallidus internus), LC (locus coeruleus), LDT (laterodorsal tegmental nucleus), MCH (melanin concentrating hormone), NE (norepinephrine), PB (parabrachial nucleus), OX-SAP (orexin-2-saporin conjugate), PPT (pedunculopontine-tegmental nucleus), REM (rapid eye movement), VTA (ventral tegmental area), SLD (sublaterodorsal nucleus), SNc (substantia nigra compacta), SNr (substantia nigra reticulata), STN (subthalamic nucleus), TMN (tuberomamillary nucleus), VLPO (ventrolateral preoptic nucleus). | | | |

**Table S2. Inputs and outputs of the basal ganglia.**

| **Structure** | **Afferents** | **Efferents** |
| --- | --- | --- |
| **Striatum** | Cerebral cortex, thalamus (intralaminar nucleus, midline and relay nuclei), SNr, SNc, GPi, GPe, STN, amygdala, hippocampus, hypothalamus, cerebellum, VTA, dorsal raphe nucleus, pedunculopontine nucleus, lateral dorsal nucleus, basal forebrain (18, 54-61) | GPi, GPe, SNr, SNc (54) |
| **GPi** | Cerebral cortex, striatum,  Thalamus (centromedian/ parafascicular nucleus), SNc, GPe, STN, dorsal raphe nucleus, PPN(1) | Thalamus (VA, VL, anterior nucleus, centromedian/parafascicular nucleus), lateral habenula, PPN(1). GPv projects to STN, hypothalamus, amygdala, dorsomedial thalamic nucleus (58) |
| **GPe** | Cerebral cortex, striatum, GPi, STN, thalamus (centromedian/ parafascicular nucleus), SNc, dorsal raphe nucleus,  PPN | Cerebral cortex, STN, striatum, GPi, SnR, nRT (54) |
| **SnR** | Striatum, GPe, STN (54) | Thalamus (VA/VL nucleus), superior colliculus, PPN (54) |
| **STN** | Cerebral cortex, GPe, SNc thalamus (centromedian/ parafascicular nucleus), PPN, dorsal raphe nucleus, amygdala, basal forebrain, hypothalamus, locus coeruleus, parabrachial nucleus, zona incerta  thalamic reticular nucleus, dorsolateral tegmental nucleus (54) | GPi, GPe, SNr, PPN, striatum, cerebellum, cerebral cortex, pontine reticular formation, basal forebrain (54) |
| Abbreviations: CM (centromedian nucleus), GPe (globus pallidus externus, GPi (globus pallidus internus), nRT (thalamic reticular nucleus), PF (parafascicular nucleus), PPN (pedunculopontine nucleus), SNc (substantia nigra compacta), SNr (substantia nigra reticulata), STN (subthalamic nucleus), VTA (ventral tegmental area), VA (ventral anterior nucleus), VL (ventral lateral nucleus). | | |

Supplementary References:

1. Eid L, Parent M. Chemical anatomy of pallidal afferents in primates. Brain Struct Funct. 2016;221(9):4291-317.

2. Sutoo D, Akiyama K, Yabe K, Kohno K. Quantitative analysis of immunohistochemial distributions of cholinergic and catecholaminergic systems in the human brain. Neuroscience. 1994;58(1):227-34.

3. Wallman MJ, Gagnon D, Parent M. Serotonin innervation of human basal ganglia. Eur J Neurosci. 2011;33(8):1519-32.

4. Hornung JP. The human raphe nuclei and the serotonergic system. Journal of chemical neuroanatomy. 2003;26(4):331-43.

5. Pollak Dorocic I, Furth D, Xuan Y, Johansson Y, Pozzi L, Silberberg G, et al. A whole-brain atlas of inputs to serotonergic neurons of the dorsal and median raphe nuclei. Neuron. 2014;83(3):663-78.

6. Sparta DR. Cartography of serotonergic circuits. 2014;83(3):513-5.

7. Ursin R. Changing concepts on the role of serotonin in the regulation of sleep and waking. In: Monti JM, Pandi-Perumal BL, Jacobs BL, Nutt DJ, editors. Serotonin and Sleep: Molecular, Functional and Clinical Aspects. Switzerland: Birkhauser 2008. p. 3-21.

8. Lyamin OI, Lapierre JL, Kosenko PO, Kodama T, Bhagwandin A, Korneva SM, et al. Monoamine Release during Unihemispheric Sleep and Unihemispheric Waking in the Fur Seal. Sleep. 2016;39(3):625-36.

9. Murray NM, Buchanan GF, Richerson GB. Insomnia Caused by Serotonin Depletion is Due to Hypothermia. Sleep. 2015;38(12):1985-93.

10. Cho JR, Treweek JB, Robinson JE, Xiao C, Bremner LR, Greenbaum A, et al. Dorsal Raphe Dopamine Neurons Modulate Arousal and Promote Wakefulness by Salient Stimuli. Neuron. 2017;94(6):1205-19.e8.

11. Schwarz LA, Miyamichi K, Gao XJ, Beier KT, Weissbourd B, DeLoach KE, et al. Viral-genetic tracing of the input-output organization of a central noradrenaline circuit. Nature. 2015;524(7563):88-92.

12. Hobson JA, McCarley RW, Wyzinski PW. Sleep cycle oscillation: reciprocal discharge by two brainstem neuronal groups. Science. 1975;189(4196):55-8.

13. Aston-Jones G, Bloom FE. Activity of norepinephrine-containing locus coeruleus neurons in behaving rats anticipates fluctuations in the sleep-waking cycle. The Journal of neuroscience : the official journal of the Society for Neuroscience. 1981;1(8):876-86.

14. Takahashi K, Kayama Y, Lin JS, Sakai K. Locus coeruleus neuronal activity during the sleep-waking cycle in mice. Neuroscience. 2010;169(3):1115-26.

15. Carter ME, Yizhar O, Chikahisa S, Nguyen H, Adamantidis A, Nishino S, et al. Tuning arousal with optogenetic modulation of locus coeruleus neurons. Nat Neurosci. 2010;13(12):1526-33.

16. Blanco-Centurion C, Gerashchenko D, Shiromani PJ. Effects of Saporin-Induced Lesions of Three Arousal Populations on Daily Levels of Sleep and Wake. The Journal of neuroscience : the official journal of the Society for Neuroscience. 2007;27(51):14041-8.

17. Hunsley MS, Palmiter RD. Norepinephrine-deficient mice exhibit normal sleep-wake states but have shorter sleep latency after mild stress and low doses of amphetamine. Sleep. 2003;26(5):521-6.

18. Yetnikoff L, Lavezzi HN, Reichard RA, Zahm DS. An update on the connections of the ventral mesencephalic dopaminergic complex. Neuroscience. 2014;282:23-48.

19. Eban-Rothschild A, Rothschild G, Giardino WJ, Jones JR, de Lecea L. VTA dopaminergic neurons regulate ethologically relevant sleep-wake behaviors. Nat Neurosci. 2016;19(10):1356-66.

20. Jackson A, Crossman AR. Nucleus tegmenti pedunculopontinus: efferent connections with special reference to the basal ganglia, studied in the rat by anterograde and retrograde transport of horseradish peroxidase. Neuroscience. 1983;10(3):725-65.

21. Benarroch EE. Pedunculopontine nucleus: Functional organization and clinical implications. Neurology. 2013;80(12):1148-55.

22. Aravamuthan BR, Muthusamy KA, Stein JF, Aziz TZ, Johansen-Berg H. Topography of cortical and subcortical connections of the human pedunculopontine and subthalamic nuclei. Neuroimage. 2007;37(3):694-705.

23. Kroeger D, Ferrari LL, Petit G, Mahoney CE, Fuller PM, Arrigoni E, et al. Cholinergic, Glutamatergic, and GABAergic Neurons of the Pedunculopontine Tegmental Nucleus Have Distinct Effects on Sleep/Wake Behavior in Mice. The Journal of neuroscience : the official journal of the Society for Neuroscience. 2017;37(5):1352-66.

24. Boucetta S, Cisse Y, Mainville L, Morales M, Jones BE. Discharge profiles across the sleep-waking cycle of identified cholinergic, GABAergic, and glutamatergic neurons in the pontomesencephalic tegmentum of the rat. The Journal of neuroscience : the official journal of the Society for Neuroscience. 2014;34(13):4708-27.

25. Van Dort CJ, Zachs DP, Kenny JD, Zheng S, Goldblum RR, Gelwan NA, et al. Optogenetic activation of cholinergic neurons in the PPT or LDT induces REM sleep. Proceedings of the National Academy of Sciences of the United States of America. 2015;112(2):584-9.

26. Petrovic J, Ciric J, Lazic K, Kalauzi A, Saponjic J. Lesion of the pedunculopontine tegmental nucleus in rat augments cortical activation and disturbs sleep/wake state transitions structure. Exp Neurol. 2013;247:562-71.

27. Fuller PM, Sherman D, Pedersen NP, Saper CB, Lu J. Reassessment of the structural basis of the ascending arousal system. The Journal of comparative neurology. 2011;519(5):933-56.

28. Qiu MH, Chen MC, Fuller PM, Lu J. Stimulation of the Pontine Parabrachial Nucleus Promotes Wakefulness via Extra-thalamic Forebrain Circuit Nodes. Curr Biol. 2016;26(17):2301-12.

29. Jones BE, Cuello AC. Afferents to the basal forebrain cholinergic cell area from pontomesencephalic--catecholamine, serotonin, and acetylcholine--neurons. Neuroscience. 1989;31(1):37-61.

30. Rye DB, Wainer BH, Mesulam MM, Mufson EJ, Saper CB. Cortical projections arising from the basal forebrain: a study of cholinergic and noncholinergic components employing combined retrograde tracing and immunohistochemical localization of choline acetyltransferase. Neuroscience. 1984;13(3):627-43.

31. Anaclet C, Pedersen NP, Ferrari LL, Venner A, Bass CE, Arrigoni E, et al. Basal forebrain control of wakefulness and cortical rhythms. Nature communications. 2015;6:8744.

32. Haas HL, Sergeeva OA, Selbach O. Histamine in the nervous system. Physiol Rev. 2008;88(3):1183-241.

33. Takahashi K, Lin JS, Sakai K. Neuronal activity of histaminergic tuberomammillary neurons during wake-sleep states in the mouse. The Journal of neuroscience : the official journal of the Society for Neuroscience. 2006;26(40):10292-8.

34. Fujita A, Bonnavion P, Wilson MH, Mickelsen LE, Bloit J, de Lecea L, et al. Hypothalamic Tuberomammillary Nucleus Neurons: Electrophysiological Diversity and Essential Role in Arousal Stability. The Journal of neuroscience : the official journal of the Society for Neuroscience. 2017;37(39):9574-92.

35. Tsujino N, Sakurai T. Orexin/hypocretin: a neuropeptide at the interface of sleep, energy homeostasis, and reward system. Pharmacol Rev. 2009;61(2):162-76.

36. Mileykovskiy BY, Kiyashchenko LI, Siegel JM. Behavioral correlates of activity in identified hypocretin/orexin neurons. Neuron. 2005;46(5):787-98.

37. Thannickal TC, Moore RY, Nienhuis R, Ramanathan L, Gulyani S, Aldrich M, et al. Reduced number of hypocretin neurons in human narcolepsy. Neuron. 2000;27(3):469-74.

38. Adamantidis AR, Zhang F, Aravanis AM, Deisseroth K, de Lecea L. Neural substrates of awakening probed with optogenetic control of hypocretin neurons. Nature. 2007;450(7168):420-4.

39. Tsunematsu T, Kilduff TS, Boyden ES, Takahashi S, Tominaga M, Yamanaka A. Acute optogenetic silencing of orexin/hypocretin neurons induces slow-wave sleep in mice. The Journal of neuroscience : the official journal of the Society for Neuroscience. 2011;31(29):10529-39.

40. Starzl TE, Magoun HW. ORGANIZATION OF THE DIFFUSE THALAMIC PROJECTION SYSTEM. Journal of neurophysiology. 1951;14(2):133-46.

41. Groenewegen HJ, Berendse HW. The specificity of the 'nonspecific' midline and intralaminar thalamic nuclei. Trends Neurosci. 1994;17(2):52-7.

42. Steriade M, McCormick DA, Sejnowski TJ. Thalamocortical oscillations in the sleeping and aroused brain. Science. 1993;262(5134):679-85.

43. Starzl TE, Taylor CW, Magoun HW. Ascending conduction in reticular activating system, with special reference to the diencephalon. Journal of neurophysiology. 1951;14(6):461-77.

44. Lu J, Sherman D, Devor M, Saper CB. A putative flip-flop switch for control of REM sleep. Nature. 2006;441(7093):589-94.

45. Weber F, Chung S, Beier KT, Xu M, Luo L, Dan Y. Control of REM sleep by ventral medulla GABAergic neurons. Nature. 2015;526(7573):435-8.

46. Monti JM, Torterolo P, Lagos P. Melanin-concentrating hormone control of sleep-wake behavior. Sleep medicine reviews. 2013;17(4):293-8.

47. Hassani OK, Lee MG, Jones BE. Melanin-concentrating hormone neurons discharge in a reciprocal manner to orexin neurons across the sleep-wake cycle. Proceedings of the National Academy of Sciences of the United States of America. 2009;106(7):2418-22.

48. Tsunematsu T, Ueno T, Tabuchi S, Inutsuka A, Tanaka KF, Hasuwa H, et al. Optogenetic manipulation of activity and temporally controlled cell-specific ablation reveal a role for MCH neurons in sleep/wake regulation. The Journal of neuroscience : the official journal of the Society for Neuroscience. 2014;34(20):6896-909.

49. Jego S, Glasgow SD, Herrera CG, Ekstrand M, Reed SJ, Boyce R, et al. Optogenetic identification of a rapid eye movement sleep modulatory circuit in the hypothalamus. Nat Neurosci. 2013;16(11):1637-43.

50. Vetrivelan R, Kong D, Ferrari LL, Arrigoni E, Madara JC, Bandaru SS, et al. Melanin-concentrating hormone neurons specifically promote rapid eye movement sleep in mice. Neuroscience. 2016;336:102-13.

51. Chou TC, Bjorkum AA, Gaus SE, Lu J, Scammell TE, Saper CB. Afferents to the ventrolateral preoptic nucleus. The Journal of neuroscience : the official journal of the Society for Neuroscience. 2002;22(3):977-90.

52. Chung S, Weber F, Zhong P, Tan CL, Nguyen TN, Beier KT, et al. Identification of preoptic sleep neurons using retrograde labelling and gene profiling. Nature. 2017;545(7655):477-81.

53. Weber F, Dan Y. Circuit-based interrogation of sleep control. Nature. 2016;538(7623):51-9.

54. Parent A, Hazrati LN. Functional anatomy of the basal ganglia. I. The cortico-basal ganglia-thalamo-cortical loop. Brain Res Rev. 1995;20(1):91-127.

55. Hoshi E, Tremblay L, Feger J, Carras PL, Strick PL. The cerebellum communicates with the basal ganglia. Nat Neurosci. 2005;8(11):1491-3.

56. Prensa L, Gimenez-Amaya JM, Parent A, Bernacer J, Cebrian C. The nigrostriatal pathway: axonal collateralization and compartmental specificity. Journal of neural transmission Supplementum. 2009(73):49-58.

57. Dautan D, Huerta-Ocampo I, Witten IB, Deisseroth K, Bolam JP, Gerdjikov T, et al. A major external source of cholinergic innervation of the striatum and nucleus accumbens originates in the brainstem. The Journal of neuroscience : the official journal of the Society for Neuroscience. 2014;34(13):4509-18.

58. Parent A. Extrinsic connections of the basal ganglia. Trends Neurosci. 1990;13(7):254-8.

59. Mesulam MM, Mash D, Hersh L, Bothwell M, Geula C. Cholinergic innervation of the human striatum, globus pallidus, subthalamic nucleus, substantia nigra, and red nucleus. The Journal of comparative neurology. 1992;323(2):252-68.

60. Wall NR, De La Parra M, Callaway EM, Kreitzer AC. Differential innervation of direct- and indirect-pathway striatal projection neurons. Neuron. 2013;79(2):347-60.

61. Parent A, Hazrati L-N, Lavoie B. The Pallidum as a Dual Structure in Primates. In: Bernardi G, Carpenter MB, Di Chiara G, Morelli M, Stanzione P, editors. The Basal Ganglia III. Boston, MA: Springer New York; 1991. p. 81-8.
